# "I'm the Momma": Using photo-elicitation to understand matrilineal influence on family food choice

**DOI:** 10.1186/1472-6874-10-21

**Published:** 2010-06-17

**Authors:** Cassandra M Johnson, Joseph R Sharkey, Alex W McIntosh, Wesley R Dean

**Affiliations:** 1 Department of Social and Behavioral Health, School of Rural Public Health, Texas A&M Health Science Center, College Station, TX, USA; 2 Program for Research in Nutrition and Health Disparities, School of Rural Public Health, Texas A&M Health Science Center, College Station, TX, USA; 3 Department of Sociology, Texas A&M University, College Station, TX, USA

## Abstract

**Background:**

Many complex and subtle aspects relating to mothers and food choice are not well understood. Mothers play a primary role in their children's food choices, but research has not specifically examined how matrilineal family members who do not reside in the same household, such as a mother's mother, aunt, or grandmother, influence the current family's food choices.

**Methods:**

Seven participants were recruited from the Household Food Inventory (HFI) Study in the Bryan/College Station, Texas. All participants completed an in-depth interview, photographed food-related activities, and discussed photographs in a follow-up in-depth interview. Interviews were transcribed verbatim from audio recordings. Transcripts were analyzed using several qualitative approaches including grounded theory to identify themes and subthemes.

**Results:**

Participants discussed the following themes relating to the influence of their mother or other female relation (Mom) on their families' food choices: Relationship with Mom, Just like Mom, 'Kinda' like Mom, Different than Mom, and Mom's Influence on Children's Food Choices. Overall, participants used the photographs to illustrate how they were similar or different to their mothers, or other female family member, as well as how their mothers either supported or undermined control over their children's food choices. The "Mom effect" or matrilineal influence of mothers, aunts, and grandmothers on a mother's food choices was omnipresent, even though Mom was no longer living with the participants.

**Conclusions:**

We found a matrilineal influence to have a residual and persistent influence on a family's food choices. This finding may be helpful for understanding the contextual elements of food choice and explaining why it is sometimes difficult to change mothers' food habits.

## Background

Mothers, whether through providing food for the home or serving as a model for eating behavior, are a primary influence on their children's food choices and health [1-4]. However, many complex and subtle aspects of mothers' influences on food choice are not well understood [5]. This study will use data from in-depth interviews and photo-elicitation, to understand how mothers make food choices for their family, which may be used to improve diet and health in families.

Mothers consider the needs of their family (e.g. children, partner, and extended family members) and other constraints, such as time and cost, as they make daily decisions related to planning meals, shopping for food, and preparing meals [6,7]. These decisions can affect a child's present and future food choices. In the present, a mother selects what foods are available at home and what meals are provided. Studies demonstrate the association of food resources in the home with children's intake of soft-drinks [8] and fruit and vegetables [9]. Evidence suggests mothers' modeling of unhealthy food choices, such as consuming sweet snacks or high-energy drinks, may influence children's intake of sweet snacks and high-energy drinks [10]. In addition, research indicates that mother shapes her child's eating habits, including self-control in eating and food preferences [11,12], which can remain with the child in the future. Studies discuss the importance of a mother's feeding style on her children's weight status [12,13]. Faith and colleagues found an association between a mother's food choices for her child, the child's compliance with her selections, and the child's body mass index [13]. Contradictory findings showed mothers made less healthy food choices for their children than they did for themselves, but research also indicated health was the most important factor when mothers were choosing foods for their children [14].

A child's mother is not the only key player in determining the child's food choices. Other family members also influence how individuals make food choices [15,16], and for women, a woman's mother can have a lasting impression on her daughter's and grandchildren's food choices. Women typically learn about food and cooking from their mothers, or others, such as grandmothers and mothers-in-law, during childhood and adolescence [17] and incorporate their mother's food practices into their own food choices [18,19]. Scholars conceptually describe how food practices are transferred from mothers to daughters, who are also mothers, through "solidarity and separation processes" [18]. Furthermore, a woman's mother can be a present influence on her family's food choices through direct interactions with grandchildren [20,21]. Studies indicate grandmothers may "undermine" a mother's control over the mother's food choices for her child [6,22].

In order to capture and understand the complex and subtle nature of how mothers feed their families, different methods are required to uncover what Mela calls the "uniquely human" aspects of food choice [23]. For example, visual methods, such as photo-elicitation, may be advantageous for examining food choice and mothers because prior work highlighted "linguistic incongruence", or the inability to describe experiences related to "feeding the family" (in interviews) with words alone [7,24]. As defined by Harper, photo-elicitation means "inserting a photograph into a research interview" [25]. Research incorporating photo-elicitation uses photographs, either taken by the participant, researcher, or from another source, as a tool to extract information related to participants' experiences [26] and "recover unarticulated aspects". In addition, participants participate in the research process by taking photographs and reflecting on the images. These methods empower participants to record, reflect upon, and discuss the "reality of their lives" [27]. However, photo-elicitation or other methods of photo interviewing have rarely been used in food choice research. Notable exceptions include a study in the American Southwest with Mexican-Americans that used photo-elicitation to examine women's diets [28] and a New York study that used photovoice to understand low-income and homeless women's access to healthy foods [29].

Despite much research on food choice, little is known about the influence of a mother's mother, or other female family member, on a family's food choices. Researchers have called for utilizing visual methods in "feeding the family" research [7,24], but there are no studies we are aware of that used photo-elicitation to examine the food choice process from a mother's perspective. Thus, this paper will use photo-elicitation with interviews to explore the personal relationship between a mother and her mother, grandmother, or other female relation, and its influence on the present family's food choices.

## Methods

### Participants

The study included seven mothers who were recruited from the 2008 Brazos Valley Household Food Inventory (HFI) study. The HFI, recruited low-income women from the Bryan, Texas area with at least one child under the age of eighteen living in the same household and included multiple in-home observations of household food resources [30]). Researchers from the HFI study recruited participants for this study by telephone. Eight mothers were originally recruited into the study, but one mother failed to arrive for the first interview or return phone calls. She was excluded from the final sample. All seven participants provided consent to participate in two audio-recorded interviews. The institutional Review Board at Texas A&M University approved this study.

This paper reports preliminary findings from a larger project aimed at understanding how food choice is operationalized through self-identification of roles and values. The project's main research questions were: 1) how do mothers balance food-related responsibilities in their families, and 2) how do they understand and address issues of healthy eating with their family? The following sections describe the materials (i.e. interview guides and photography assignment) used for data collection.

### Study Materials

#### Interview 1

A 6-item interview guide was iteratively developed to elicit mothers' personal experiences related to the food choice process. Each 30 to 45 minute in-depth interview included the following six items: choose a typical week day and describe your activities; pick a weekend day and describe your activities; what types of things affect the way you think about food and the choices you make about food; what do you teach your kids about food; do you remember a time where you disagreed with your family about what to eat; as a mother, what have you learned about feeding you family over the years?

#### Photo-elicitation activity

To reveal information inaccessible in interviews alone, a photography assignment and second interview using photo-elicitation were used. The photo-elicitation activity was introduced after completion of the first interview. Each participant was provided with written protocol, a verbal explanation, and hands-on demonstration of the disposable camera. The protocol began with an introduction and description of purpose based on existing studies using photovoice or photo-elicitation methodology, with an emphasis on health, women, and mothers. Then, the photography assignment was outlined with step-by-step instructions. Each participant was instructed to take at least 15 photographs for the assignment and use the remaining exposures for personal photographs. A demonstration camera was provided to ensure that the participants were comfortable with the activity.

#### Interview 2

An interview guide for the photo-elicitation interview included story prompts developed from the literature [27-29,31,32]. Several studies used Shaffer's SHOwED technique for discussing photographs [27,32-34]. These prompts were used: what do you see in this picture; and what does this picture mean to you? Other story prompts included: what's happening in this picture; why did you take this picture; what is missing from this picture; when was this picture taken; what was special about this picture; what does the picture symbolize; how does this picture make you feel; did you set-up this picture and why; and what were you doing when this picture was taken? Each interview was designed to take 30 to 45 minutes.

### Data Collection

Data collection (both interviews) took place in the participant's home; were conducted by a team of two trained researchers; and were audio recorded. During the initial visit, participants provided written consent and completed a 1-page paper survey prior to the in-depth interview. The survey was interviewer administered and included sociodemographic characteristics (e.g. age, education, marital status, household composition, and household income), transportation, nutrition program participation, and food accessibility. During the first interview, the first author used the interview guide and conducted the interview, while the other researcher served as observer and wrote detailed field notes related to the interview, participant, and surroundings. On average, the in-depth interview lasted 48 minutes (range 25-73 minutes). At the end of the interview, each participant was provided a 27-exposure disposable-camera, verbal and written instructions (i.e. protocol) for the photography assignment. In addition, participants were given an opportunity to practice taking photographs with a demonstration camera and reminded that one complete set of the photographs would be given to them at the second visit. Approximately five to seven days after the initial interview, cameras were collected from all participants and photographs were developed. For each camera, one compact disc with digital photograph files and two sets of hard copy photos were created.

At the beginning of the second interview (approximately 7-10 days after the first interview), the first author asked each participant to describe her experience with the photography activity. The participant was given her set of photographs and asked to select approximately five photographs for discussion. For each photograph, the participant was guided by the story prompts outlined in the interview guide and asked to elaborate on the photograph. In addition, participants were asked to title each photograph.

After discussing participant-selected photographs, each participant was asked to discuss additional photographs selected by the first author. On average, the photo-elicitation interview lasted 34 minutes (range 22-44 minutes). Each participant received a $60 honorarium for participation in the project.

### Data Analysis

Interview audio files were transcribed verbatim into Word documents; all audio recordings were reviewed for accuracy of transcription. Photographs and photograph titles were inserted into the photo-elicitation transcripts for utilization during analysis. A pseudonym (i.e. Vicki, Pat, Lola) was assigned to each participant in order to personalize the interviews.

Analysis was guided by several qualitative approaches including grounded theory [35-37], and Sift and Sort: Think and Shift [38]. Transcripts were analyzed manually using an iterative process of recording, reflecting upon, and reviewing observations. All transcripts were read multiple times; audio recordings were reviewed to "pick up" on additional observations, which may have been hidden in text. Living documents, or memos, were generated and continually updated in this process to understand and then interpret participants' responses [37,38]. For example, one document answered these questions: what did I learn from this participant's transcript; and why is this participant important [38]. This process revealed an overarching theme related to the influence of a mother's mother, aunt or other female family member, referred to as Mom. After this discovery, preliminary findings were shared with the project team for discussion and peer debriefing. Then, transcripts were coded using inductive codes.

The most salient codes, such as participant's "Relationship with Mom", "Just like Mom", "'Kinda' like Mom", "Different than Mom", and "Mom's Influence on Children's Food Choices" were identified as themes. Text segments related to these codes were extracted from transcripts and compared by participant, by interview, and for both interviews to determine how this matrilineal influence affects mothers' food choices. In addition, mothers' differing attitudes (i.e. positive/negative, pleased/resentful) towards the mother-daughter relationship, extent of food and eating practice replication, or influence of their mother on their children's food choices were also noted as subthemes.

## Results

### Participant characteristics

Participants' characteristics are shown in Table 1. The seven mothers were 26 - 42 years old. Most of the participants were white Anglos (non-Hispanic white); two participants were Hispanic. Each participant had at least one child living with them full-time, and five participants had more than one child living with them at home. All of the households were composed of only the mother, her partner or husband, and children; no grandparents or extended family members lived with the participants at the time of the interviews.

**Table 1:** Participant's Survey Data

	Vicki	Pat	Lola	Paris	Liz	Sunny	Gladys
**Age**	26	39	33	32	37	37	42
**Education (highest completed)**	Not reported	12^th ^Grade	12^th ^Grade	GED (some college and technical school)	9^th ^Grade	12^th ^Grade	12^th ^Grade
**Income**	$30 k-$35 k	$20 k-$25 k	$16 K-$19.9 k	$20 k-$25 k	< $10,000	$36 k-$39 k	$20 k-$25 k
**Household composition**	Mother & 1 child	Mother, Partner & 2 children	Mother, Husband, & 4 children	Mother, Husband, & 1 child	Mother & 5 children	Mother, Husband, & 4 children	Mother, Husband, & 3 children
**Food assistance programs**	N/A	Reduced or free school lunch	Food Stamps (Lone Star Card) **and **Reduced or free school lunch	Free breakfast for children **and **Reduced or free school lunch	Food stamps (Lone Star Card)	WIC, Free breakfast for children **and **reduced or free school lunch	Free breakfast for children
**Car ownership**	Owns Car	Owns Car	Owns Car	Owns Car	Owns Car	Owns Car	Owns Car
**Health Conditions**	N/A	N/A	Obesity	N/A	N/A	Diabetes (extended family)	N/A

During the analysis, major themes and subthemes were recorded and are presented in Tables 2-6. The major themes included: Relationship with Mom, Just like Mom, 'Kinda' like Mom, Different than Mom, and Mom's Influence on Children's Food Choices. The first theme presents the participants' general relationship with their mothers and provides context for understanding the matrilineal influence on family food decisions. Remaining themes discuss the participants' food and eating habits in relation to their female family members' food choices and lastly, how Mom (grandmother) influences the children's (her grandchildren) food choices.

**Table 2:** Theme: Relationship with Mom

Theme	Relationship with Mom
**Definition**	Participants describing the mother-daughter relationship, including attitudes toward Mom (e.g. mother, grandmother, aunt).

**Representative Quotes**	"Yeah, my aunt...If I don't know som'n, I'll call her." -Gladys "...My mom does not spend much time at our house at all..." -Sunny "...She [mother] doesn't listen so it doesn't-...I'm like, 'You're feeding them too much', you know. 'They're eating too much snacks.'..." -Pat

**Subthemes**	Established relationship (positive attitude toward Mom); Disconnected relationship demonstrated (neutral attitude toward Mom); Strained relationship (negative attitude toward Mom)

**Table 3:** Theme: Just like Mom

Theme	Just like Mom
**Definition**	Participants incorporating Mom's food practices with her children in her own home

**Representative Quotes**	"Well, uh, she [mother] just uh showed me like, if I give them vegetables, uh, include dem on the meals, it can make healthy and uh, they can grow better, is what my mom says." -Lola "Oh my mom, I would just watch her. But she didn't like us in the kitchen gettin' in the way. Like I do my kids, but um..." -Pat

**Subthemes**	Pleased to be Just like Mom; Resentful to be Just like Mom

**Table 4:** Theme: 'Kinda' like Mom

Theme	'Kinda' like Mom
**Definition**	Definition: Participants sharing how they partially reproduce Mom's food habits, mostly with special and holiday meals

**Representative Quotes**	"You know I didn't really learn nothin', and I regret that...She's [mother's] deceased, but I regret that. You know, because my Auntie had to come aroun' an' teach me like how cook...Thanksgiving and Christmas y'know." -Gladys "So far, everything that my mom has cooked in her lifetime...like the meatloaves...I have cooked behind her...I have never really had no problem." -Liz

**Subthemes**	None

**Table 5:** Theme: Different than Mom

Theme	Different than Mom
**Definition**	Definition: Participants rejecting Mom's food habits in favor of something different, usually to reflect more health-conscious values

**Representative Quotes**	"...My mom...It was so easy for her to run through McDonald's and be done with it. Two happy meals-kids are fed. And so that's pretty much how we-, we grew up and went through school...It's just recently changed as I've gotten out on my own, and I know, I mean I can balance what's right for you and you know not so good for you."-Vicki "Yep, they [children] don't care for vegetables, but see-. I grew up like that...I really don't care. I just started makin' myself eat vegetables cuz I usedta didn't eat 'em at all. So I try to...throw in the spinach...throw in the uh, greens, and throw it all in there you know..." -Gladys

**Subthemes**	None

**Table 6:** Theme: Mom's Influence on Children's Food Choices

Theme	Mom's Influence on Children's Food Choices
**Definition**	Participants' sharing attitudes and experiences regarding Mom's influence on children's food choices, mostly through direct interactions with grandchildren.

**Representative Quotes**	"Well, my mom is like most, the person that sometimes watch for me, so I don't have to tell her what to...She's more, like picky than me...You have to eat this! Ha ha! (laughing)." -Lola "...One thing I won't let him [son]...those energy drinks. I'm like, 'No, you don't need that, and you're not gonna drink that.' And it's a battle cuz he will want one, or his grandma will buy one for him. I'm like, 'No, that's not good for you right now.' So..." -Pat

**Subthemes**	Pleased with Mom's influence on children; Frustration with Mom's influence on children

In total, participants took 10-18 photographs, including ones of themselves or family members preparing or eating food, plated foods, and foods on counters or in storage. In addition, photographs highlighted family meals, special foods, such as desserts, and foods for certain occasions (i.e. fast food on a Friday night, party foods, and dinner with guests). Others included more supportive images of kitchen appliances, recipe books, and food stored in refrigerators, freezers, and cupboards. Participants presented a few photographs depicting more subtle aspects such as foods from someone else (i.e. co-worker, family member) and foods prepared or eaten away from home. Photographs selected for inclusion serve to illustrate how a participant's mother, or other female family member, influenced the participant's food choices. Some photographs discussed below were not included due to image quality. The photographs' titles, named by the participant, are provided in quotation marks in text.

### Relationship with Mom

Participants spoke about their relationships with their mothers, aunts, and in some cases, their grandmothers, which was captured by the theme "Relationship with Mom". Three subthemes were identified where the participants described established, disconnected, and strained relationships with their mothers or other female family members.

#### Established relationship with Mom

Typically, participants had established relationships with their mothers, grandmothers, or aunts, demonstrated by a positive attitude toward Mom. For example, Liz, mentioned how her mother was coming down the next weekend. During the second interview, Liz took a photograph ("Granddaughter & Grandma Snack") of a bag of snacks, which were purchased for an outdoor concert that Liz, her youngest daughter, and Liz's mother were attending. Other participants also demonstrated a positive relationship with Mom. For example, Vicki described how she and her son go to her parents' house for weekend barbecues and to restaurants with her grandmother. During the photograph discussion, Vicki happily recalled a photograph ("Him and Nana") of her son baking chocolate chip cookies with her mother. Although Gladys' mother was deceased, Gladys spoke fondly of her aunt and how she relies on "Auntie" when she is not sure about something. In the second interview, Gladys said her aunt eats with them "all the time" when discussing her photographs, suggesting closeness between her and her "Auntie".

#### Disconnected relationship with Mom

Two participants expressed more neutral attitudes towards their mothers. Compared to other participants, Sunny and Paris's relationships with their mothers did not seem to be connected. During the interviews, they did not reveal much about their mothers, except when discussing food and eating habits specifically. For example, Sunny mentioned her mother a few times when talking about what she had learned as a mother or how she wanted different rules for her children. However, generally and compared to others, Sunny rarely brought up her mother in conversation and they did not seem to spend much time together. Despite this, Sunny talked about how she will call her mother to see if her mother "did the same things" with her children. Another participant Paris hardly spoke in general terms about her relationship with her mother in either interview. Instead, she contrasted foods and meals from her childhood with food choices she makes now for herself and family.

#### Strained relationship with Mom

Only one participant, Pat expressed a negative attitude towards her mother; the mother-daughter relationship seemed strained. In the first interview, Pat said "...it's a battle. It is kinda hard, reprogramming 'em, but I don't have to pay for day-, daycare, so I don't mind. You know, what can I do?" Although Pat's mother helps her daughter by keeping her children during the day, Pat seemed to have an overall frustrated tone when talking about her mother, especially when explaining how her mother does not listen to her regarding her children's food choices. In the second interview, Pat expressed similar levels of tension towards her mother, and she used photographs to elaborate how she struggles with her mother regarding what to feed her kids.

### Just like Mom-Their mothers' daughters

The theme "Just like Mom" connected mothers' descriptions related to being their mothers' daughters and the replication of their mothers', aunts', or grandmothers' food and eating practices. Within this theme, there were two subthemes, pleased and resentful, determined by the participant's attitude towards being her mother's daughter and having similar food practices to Mom.

#### Pleased to be Just like Mom

In the first interview, Lola indicated she was pleased in carrying-on cooking habits learned from her mother and grandmother. In addition, Lola learned about the importance of eating vegetables and maintaining health, from her mother. For example, Lola shared, "Well, uh, she [mother] just uh showed me like, if I give them vegetables, uh, include dem on the meals, it can make healthy and uh, they can grow better, is what my mom says." Lola talked about how she has incorporated some of her mother's rules in her family such as: always having a vegetable and having her daughters eat the vegetables first. In her words, Lola encourages her daughters to eat vegetables because "...*Como se dice? Que te hace mas saludable*...." Her daughters quickly translated mother's rationale for providing vegetables by declaring together, "It makes us more healthy!". In her family, Lola passes on what she learned from her mother to her daughters. She explained, "It's what I tell them. Ha! Ha! Ha! (laughs) And it's what my mom used to tell me."

**Figure 1:**
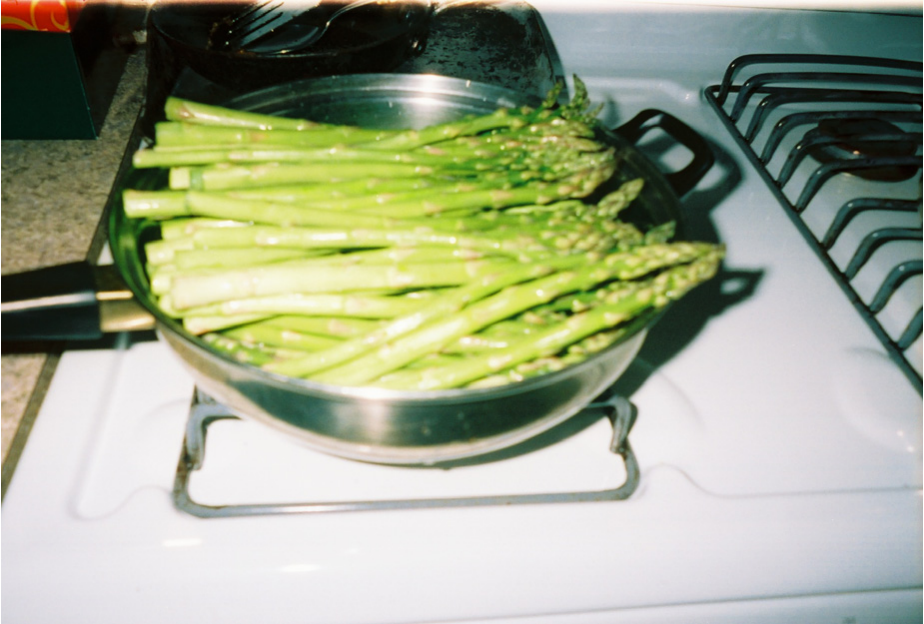
**"Mom's favorite"**. This photograph was taken during the week and selected by Lola; she named this photograph "Mom's favorite" because she likes asparagus ("I ate most of it") and made it for her family. As Lola described, "I put some on their plate, but they, they afraid to try it, but they just taste one and-." Even though she knows her children might not like asparagus at first, she wanted them to try the vegetable. This practice of providing vegetables and teaching her children to eat vegetables was learned from her mother-Lola, Interview 2, 2009.

In the second interview, Lola's mother was a present influence, and the meals depicted were often connected to Mom. For instance, Lola used a photograph called "Saturday Dinner" of her family gathered around the table to eat quesadillas, or *sincronizada*, with ham and cheese to describe how she makes foods that her mother made for her. "...I just want...a little bit more that...kind of foods that we eat 'an, um, those are some like my mom used to make for me (laughing), and uh that's why I pick...that picture..." A different set of photographs demonstrated how Lola prepares balanced meals ("The 'Goodest' Soup") and vegetables ("Mom's Favorite") for her family. In "The 'Goodest' Soup" photograph, Lola said how the photograph shows how she combined vegetables (carrots, cabbage, and carrots), beef, and pasta together to "give them most of the...nutrients..." She chose the photograph to "...show you that we can...try to balance the, what we ate." For the photograph "Mom's Favorite" (Figure 1), Lola used the photograph to illustrate an example of how she incorporates her mother's food practices into her own family's food choices. Although Lola learned about the importance of eating vegetables from her mother, she also makes additional dishes, which her mother prepares, including *arroz con leche *and oatmeal. For example, Lola showed a photograph of a dessert *arroz con leche*, or rice with milk, which she prepared especially for her "picky" daughter. During the interview, her daughters served a sample of *arroz con leche*.

#### Resentful to be Just like Mom

Not all participants were pleased to carry on Moms' food and eating practices, necessitating this subtheme defined by a participant's resentful attitude toward being her mother's daughter. During the first interview, Pat seemed to do things very much as her mother did. In her quotation below, Pat revealed how she replicated many of her mother's food practices for her family, such as making quick, ready-to-eat foods for her family, not allowing her children in the kitchen, and struggling to encourage her children to eat different foods, especially vegetables.

Oh my mom, I would just watch her. But she didn't like us in the kitchen gettin' in the way. Like I do my kids, but um...My mother didn't raise us on Mexican foods...but our relatives did, so you know we were excited to get the homemade flour tortillas and um, all that stuff, but...I don't make tortillas for my family. Ha ha! It's too much work [Pat].

However, her comment, "like I do my kids" illustrated her frustration in being her mother's daughter. A tension is also illustrated here: "I think it's [cooking] something that, that I developed more because if we tell my mom, 'Why didn't-?'. Like if we didn't wanna eat it, then she'd say, 'Okay'...Um, and I wanted to kinda be different from her....." From this quotation, Pat explains how she wants to differentiate herself from her mother by encouraging her children to try different foods, especially healthy foods and vegetables.

During her second interview, Pat explained how her son will occasionally help set the table, but he is not allowed to help cook except for packages of Ramen soup. This response provided evidence of Pat's replication of her mother's food practices. Earlier Pat shared how she wanted to be different from her mother and have her children try different foods particularly vegetables, but throughout her interviews she indicated an inability to differentiate herself from her mother. For example, when Pat's son wanted something "heavy" for breakfast like Spaghetti O's, Pat tried to tell him "You don't need that" only to later tell him, "Okay, go ahead". One photograph titled "Chef-Boy-are-Breakfast" captures Pat's conflict between encouraging her son to consume new and healthy foods and not "making him starve". This photograph highlighted Pat's similarity and her resentment towards her mother.

### 'Kinda' like Mom

Participants who did not generally reproduce their mothers' food and cooking habits but still made some of their mothers' signature or holiday dishes were described by this theme "'Kinda' like Mom". Gladys and Liz both expressed pride and pleasure in being able to carry-on some of Mom's meals. In the first interview, Gladys spoke of learning certain dishes from her mother (i.e. fried chicken and pork chops) and aunt (i.e. holiday meals):

...My Auntie had to come aroun' an' teach me like how cook...Thanksgiving and Christmas y'know...She [mother] taught me how ta fry chicken and pork chops. She [mother] taught me how to do alot. Just the main dishes like on Thanksgivin' and Christmas n'Easter an' stuff...I didn't learn from her because I didn't want to. Cuz I didn't wanna cut them onions up [Gladys].

Although Gladys was regretful about not learning as much from her mother about cooking, her mom "taught her how to do a lot" including how to prepare signature dishes like fried chicken and pork chops. When her mother was no longer around, Gladys's aunt taught her how to make holiday meals for Thanksgiving and Christmas. With her own daughters, Gladys brings them into the kitchen and teaches them how to make food choices and prepare meals.

In the second interview, Gladys used photographs of a Sunday lunch to show how she has continued the food habits of her mother and her aunt and how this matrilineal influence was present in her family's food choices. The photographs showed her husband, children, and aunt enjoying a meal of fried chicken and spaghetti. When describing the photographs, Gladys said, "my mom taught me everything, but, when my momma passed on she [aunt] like stepped in." Her photographs showed an example of a meal that Gladys learned from the female relatives in her family and she currently makes for her family.

As a teenager, Liz was married and having children. Therefore, she did not learn how to cook from her mother; she "took a little bit of home-making in school". Yet, despite different circumstances, she was also pleased to make dishes her mother also prepared. In her first interview, Liz shared how she made her first Thanksgiving meal on her own and "everything turned out". However, later, she expressed pride in being able to make the same dishes as her mother. Liz said, "So far, everything that my mom has cooked in her lifetime...like the meatloaves...I have cooked behind her...I have never really had no problem." Again, similar to Gladys, Liz is passing on what she learned from her mother and her own food practices to her oldest daughter.

### Different than Mom

The theme "Different than Mom" was used to describe how some participants talked about their reluctance to carry-on certain food and cooking habits acquired from Mom. Three participants, Vicki, Paris, and Sunny, shared how they more or less rejected what they learned from their mothers in favor of different food choices. In addition, they seemed pleased with their decisions to deviate from being their mothers' daughters.

In the first interview, Vicki seemed to describe her food choices by contrasting them to her mother's. For example, Vicki explained how her mother would "run through McDonald's and be done with it. Two happy meals-kids are fed". However, when recalling her own food choices, Vicki described herself as knowing how to "balance what's right" and at McDonald's, choosing apple wedges for her son and a grilled chicken sandwich for herself. In addition, Vicki talked extensively about her family's medical history, such as her grandmother's fatal heart attack and her mother's scare with her high blood pressure. As a single mother with career and school demands, she realized the downside to her hectic lifestyle, characterized by irregular, unhealthy food choices, and decided to make a life change for herself and her son. At one point, Vicki said:

...I wish my mom would have...done a little bit of a better job of feeding my sister...well me and my sister...because my sister-she's overweight...I mean she's...really battling with it. And I think if it would have been addressed when we were younger, then maybe it could have prevented her over-weightness right now. So that's my goal as I've addressed. Okay, you know, this is what I have to do to change my eating habits, and for the sake of him, because I don't want him [son] to...there are children that are overweight...It's not necessary, it can be addressed. Parents just need to make um the choice to you know provide healthy food for their children [Vicki].

Here, Vicki seemed regretful when describing her mother's food choices for her and her sister and the consequences. Further, the above quotation illustrates how Vicki rejected her mother's food and eating practices by providing healthy food choices and preventing future weight problems for her son.

Throughout the second interview, Vicki used photographs to highlight how she was not her mother's daughter, in terms of food and eating habits. For example, Vicki showed photographs of her grocery items, including "good protein" like lean turkey burgers, low fat yogurts, and frozen vegetables, to describe her and her son's weekday meals and snacks. Several of her pictures depicted her son eating fresh fruit with breakfast or for a snack. When sharing her food choices throughout the day, Vicki proudly detailed her pre-planned and healthy meals. However, she added her mother "doesn't buy that much" at the grocery store because she does not cook often, which emphasized the differences between mother and daughter. From both the conversation and photographs, Vicki's food choices were a departure from her mother's food habits and especially her fast food dinners.

Both Vicki and Paris shared a strong health focus. In her first interview, Paris described her attempts to do things differently and healthier than her mother.

I think that it's gotten a lot healthier...I'm from Texas, and my mom used to eat really heavy meals, so that's what I started out doing-good tasting, heavy...food. But I've learned, you know, you don't have to do that. It's not really good for you....You really need to have vegetables at every meal...[Paris].

**Figure 2:**
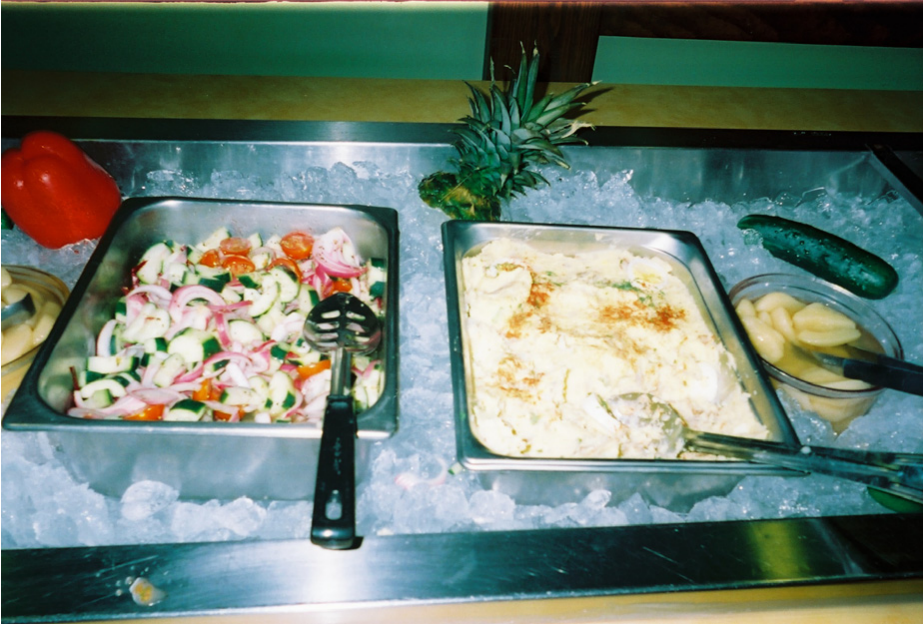
**"Choose to eat bad, Choose to eat healthy"**. This photograph was taken and chosen by Paris. Paris also titled the photograph. She shared how she learned to do things differently than her mother; she chooses healthy foods for herself and her family. This photograph showed two options in a lunch buffet-1) fresh chopped salad with cucumbers and tomatoes on the left and 2) creamy potato salad on the right. In her words, "I did not eat these mashed potatoes. It's...But I did eat some of these cucumbers and a salad..."-Paris, Interview 2, 2009.

**Figure 3:**
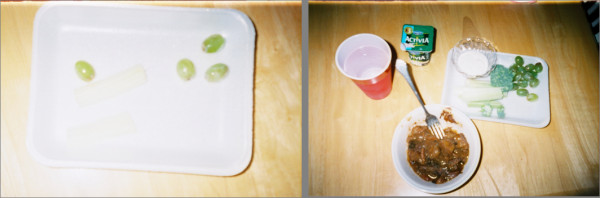
**"Before" and "After-Don't believe in cleaning your plate"**. Paris took these photographs in her home. Photographs were selected and named by Paris. The photograph on the left "Before" illustrates her lunch-pot roast with vegetables, fresh vegetables, grapes, yogurt, and water. The photograph on the right "After" shows two pieces of celery and four grapes left on her plate. She explained, "The reason I took a photograph of that's cuz you don't... I don't believe in cleaning your plate...Um, I was raised that way...And I think it's wrong and cause, causes people to be overweight. If you're raised that way, it causes you to get in the habit of cleaning your plate..."-Paris, Interview 2, 2009.

The quotation above is a good example of how a daughter may seek to separate herself from her mother's food practices. With Paris, her current food choices reflected a strong health motivation, as evident in her statement, "You really need to have vegetables at every meal". In the second interview, although she did not specifically mention her mother's influence, she used the photographs to illustrate day-to-day examples of her healthy ways of cooking and eating. Paris's responses and photographs indicated that now she eats a variety of vegetables regularly and prepares vegetables for her family. For a group of photographs titled "Choose to Eat Bad, Choose to Eat Healthy" (Figure 2). Paris explained what she would and would not eat at a buffet restaurant for lunch. Paris was matter-of-fact as she described choosing to eat the healthier options, such as cucumber and tomato salad, while skipping high-fat dishes such as fried chicken and potato salad. In addition, Paris also took before and after photographs "Before" and "After-Don't Believe in cleaning your plate" (Figure 3) to demonstrate how she abandoned her mother's rule of "cleaning her plate" at meal time although she was "raised that way".

A third participant Gladys also mentioned not growing up with vegetables. In the first interview, she said:

Yep, they [children] don't care for vegetables, but see-. I grew up like that...I really don't care. I just started makin' myself eat vegetables cuz I usedta didn't eat 'em at all. So I try to...throw in the spinach...throw in the uh, greens, and throw it all in there you know...And they pretty much all eat it [Gladys].

Although she was not accustomed to eating vegetables, Gladys started doing things differently than her mother to teach her kids the importance of eating vegetables and being healthy. In her own words, "...I try to sneak in (whispers) vegetables ...tell 'em, it's good for em. It's better for your health and stuff..."

In the same rejection of the "clean your plate" rule, Sunny also spoke about discarding her mother's rules about food and eating. During the first interview, Sunny said:

...We don't do anything like that [rules] only because there are some times where it's some foods that they don't like. And we grew up where we were forced to eat that which we did not like, and couldn't leave the table until it was done. And I won't do that to my kids...[Sunny].

In the preceding segment, Sunny articulated how she and her husband will make foods and vegetables their children like to eat instead of implementing her mother's rules, As a mother, Sunny tries to "make sure that they're putting into their bodies what they need to keep going", while providing her children foods they like to eat. Although Sunny did not directly mention her mother's influence in the second interview, she talked about various photographs where her children's individual food preferences determined the particular meal. For example, one photograph ("Her Daughter's Favorite") showed two of her younger children sitting around the table for her daughter's favorite meal-hamburgers and corn, and another photograph was of her daughter smiling with two ears of corn on her plate.

### Mom's influence on Children's Food Choices-Support vs. Sabotage!

Although this theme was not the most dominant, "Mom's influence on children's food choices" was essential for understanding how the participant's mother may directly influence her children, or Mom's grandchildren. Two participants, Lola and Pat, indicated how their mothers either supported or sabotaged, respectively, the food choices they made for their children.

#### Pleased with Mom's influence

With an established mother-daughter relationship, Lola shared how her mother supported the healthy food choices Lola encouraged in her daughters. During the first interview, Lola explained, "...my mom is like most, the person that sometimes watch for me, so I don't have to tell her what to...She's more, like picky than me...You have to eat this! Ha! ha! (laughs)." In this quotation, Lola seemed happy with her mother watching her children because her mother is more watchful with her daughters' food choices and considers eating healthy very important. The positive, reinforcing nature of Lola's mother in Lola's food choices for her children is opposite to Pat's mother, who undermined her daughter's food choices.

#### Frustration with Mom's influence

Pat's mother seemed to undermine her daughter's control over her children's food choices. During the week, Pat's mother will watch her son and daughter. Although Pat will prepare something to eat for her mother to give to her children, Pat shared her mother is "always buying them French fries". Throughout her interview, Pat expressed resentment towards her mother, because her mother watches her children, eliminating the need for child care, and provides her children unhealthy foods such as French fries and energy drinks. As a result, Pat spoke extensively about her frustration with her mother and how her mother "doesn't listen". In the following quotation, Pat articulated the conflict between her and her mother related to her children's food choices:

...She doesn't listen so it doesn't-...I'm like, 'You're feeding them too much', you know. 'They're eating too much snacks.' If she says, 'Okay, I'm not gonna feed 'em', then...they'll load up on snacks, and then they're not hungry for dinner. And...when it's time to go to bed, then they're hungry, and it's a battle...But she'll buy them McDonald's French fries, and she'll pick 'em up from school, and they'll eat that...that's too much every day, that's too much for them...[Pat].

**Figure 4:**
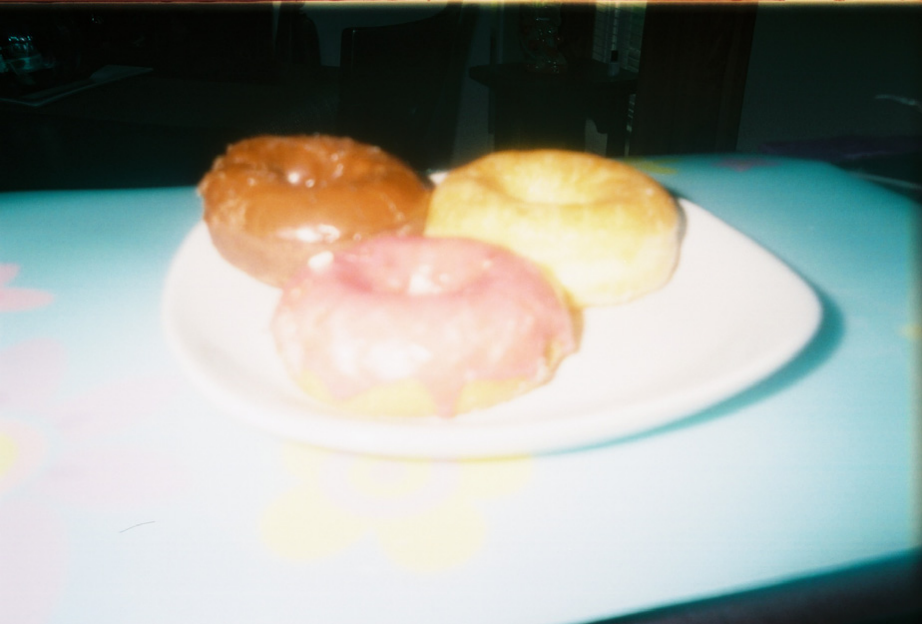
**"Grandma's treats"**. This photograph was taken in Pat's home. Researchers selected this photograph to discuss, but Pat provided the title. Throughout Pat's interview, she described frustration with her mother, especially in how her mother "feeds" her children. Pat recalled, "And I had made 'em like a big breakfast-sausage, eggs and potatoes and all that stuff. And then she came over, my mom came over and she brought donuts. And they just sat there and sat there cuz they weren't hungry..." This photograph of three iced donuts illustrates grandmother undermining Pat's control over her children's food choices-Pat, Interview 2, 2009.

In the second interview, Pat provided more details regarding her mother's influence on her children's food choices, such as how her mother also brings unhealthy foods, such as donuts, into her home. Figure 4 ("Grandma's Treats") was selected by the researchers to discuss, but serves as an example of the extent her mother undermines Pat's control over her children's food choices. Pat retold the story of her mother bringing donuts for her children, and added she does not buy donuts regularly. From Pat's perspective, her mother's disregard for Pat's food choices was frustrating because her children were full and did not want the donuts. In the donuts example, Mom, Pat's mother, brought in sweets that directly affected her grandchildren's food choices.

## Discussion

This investigation of matrilineal influence on food choice within a family contributes to the understanding of mothers' mothers, aunts, and grandmothers in families' food choices. Most importantly, this study found that the Mom effect, or the residual and persistent influence of a mother or other female relation on a daughter's family food choices, to be a major influence on her daughter's family's food choices, even when the mother is no longer omnipresent. Results provide valuable social context for understanding some unknowns in how mothers navigate the food choice process. Although a few studies have mentioned the role of a mother's mother on the (daughter's) family's food shopping, cooking, and eating practices, this qualitative investigation focused exclusively on the matrilineal influence on food choice and included other influential female family members, such as aunts and grandmothers. The strong connection between a daughter, Mom and their food habits is supported by other studies [17-19].

In addition, this paper provides a novel way for understanding the day-to-day realities of the food choice process using photo-elicitation and sequential interviews. In 2007, photo-elicitation was used to study the context of diet intake in Mexican-American women [28]. Recently, Valera et al. used Photovoice to capture women's experiences of access to healthy foods in New York [29]. However, this study appears to be the first to use a participatory visual method, such as photo-elicitation, to explore the food choice process from a mother's perspective and use two sequential individual interviews. The main advantages of combining photo-elicitation with sequential interviews included increased rapport with participants, mutual investment in project, detailed accounts of food and eating experiences, and opportunities to clarify or elaborate on responses and experiences from the first interview, in their own words.

Several major themes focused on the participants' food choices and habits in relation to their mothers (e.g. "Just like Mom", "'Kinda' like Mom", and "Different than Mom"). Overall, the results here are consistent with the Norwegian study [18] . Bugge and Almas described how dinner habits were transferred from mother to daughter by "solidarity and separation processes" because the daughter wants to both carry on her mother's practices and distinguish herself from her mother [18]. Others have also described the mother-daughter relationship as evolving over time and moving through solidarity and separation processes [39,40].

In this sample, the matrilineal influences occurred through two different processes: 1) Mom (i.e. mother, aunt, grandmother) indirectly influenced the participant by having her food and eating habits remain with participant, mainly as result of upbringing, and 2) Mom directly influenced the participant through interactions with the participant's children, her grandchildren. This study reinforces the findings of the literature on intergenerational influences, which indicates a grandmother may influence her grandchildren's food choices in the following ways: by advising the mother on child feeding; providing foods for her grandchildren; and undermining mother's control of her children's food choices [6,22,41]. This analysis provides additional refinement for understanding intergenerational influences on food choice, and specifically how a grandmother may affect her grandchildren's food choices. Finding the participant's mother played such an important role in the current mother's food choices makes sense given the maternal grandmother, followed by a maternal aunt, typically provide the most caregiving in a family [42].

Regarding the theme Just like Mom, this finding is consistent with other studies where daughters had a "strong bond to their own mothers' practice" [18]. Pashos and McBurney describe how more caring is done by the mother's side of the family, which bolsters the role of a mother's mother or other female family member on family food choice [43]. The connectedness of mothers and daughters and food makes sense based on literature describing how identities of mothers and daughters are intertwined [39].

For the participants who rejected their mothers' ways, there are different reasons why the daughters chose to do things differently. Typically, they wanted to make more healthy food choices for their own family for both health promotion and disease prevention reasons. A daughter's rejection of Mom's food and cooking habits could also be related to generational differences between the mother and daughter including new ways to be a woman, new gender roles, and new family relations [18]. Bugge and Almas found that their participants used dinner to "rebel against the codes and rules" of their youth [18].

Based on observations of this sample, the separation and solidarity processes occur within a certain social context. One interesting observation is that even in the cases where the participants replicated their mothers' habits, it was not a simple replication. For example, in some instances, participants have carried on their mothers' special dishes or holiday meals. In other examples, the participants incorporated their mothers' rules into their families and used them to teach their kids about food and eating. Also, the participants reproduced their mothers' ideas about health such as eating vegetables for healthy development. The incorporation of food practices from childhood may be an "important part of the social reproduction of family identities across generations" [18]

Lastly, grandmother's support or undermining of a mother's food choices for her children was another theme. Findings from this study indicate grandmothers may either support and encourage their daughter's healthy food choices for her children or counteract her food choices. This is partly supported by studies that have noted how family members, especially grandparents, can influence their grandchildren's food choices, by undermining the mother's control [6,22].

This study provides insights into the lives of this group of participants, their families, and food choices. However, these reported findings speak only to the particular mothers in this study. Though the results may highlight the importance of a mother's mother or other female relative on her family's food choices, the results are not generalizable to all mothers.

## Conclusion

To our knowledge, this is the first study to apply sequential interviews with photo-elicitation in food choice research. Therefore, this work extends existing work with a novel application of photo-elicitation and detailed methodology for using sequential interviews with photo-elicitation in a context-centered food choice study. Finally, while there has been substantial research on environmental factors affecting food choice, less attention has been devoted to influences within the family. Similar to other studies, these results also show a strong connection between daughters, mothers, and food habits [17-19]. Findings from this study underscore the importance of a matrilineal influence on family food choice, which adds an additional level of complexity to understanding what foods are purchased, prepared, and consumed on a daily basis.

This paper provides a social context for understanding both indirect and direct processes in which a mother's mother, or other female family member, influences the family's food choices. The pronounced effect of this matrilineal influence on a current family's food choices may have additional implications for understanding food and eating behaviors in a family context, particularly for interventions aimed at mothers to improve a family's diet and health.

## Competing interests

The authors declare that they have no competing interests.

## Authors' contributions

CMJ served as lead author, participated in the planning of the study and manuscript, and carried out the analyses. JRS participated in the design and coordination of the study and helped in planning, analyzing, drafting, and finalizing the manuscript. WRD and WAM participated in the interpretation of the data. All authors read and approved the final manuscript.

## Pre-publication history

The pre-publication history for this paper can be accessed here:

http://www.biomedcentral.com/1472-6874/10/21/prepub
